# No Evidence for XMRV Nucleic Acids, Infectious Virus or Anti-XMRV Antibodies in Canadian Patients with Chronic Fatigue Syndrome

**DOI:** 10.1371/journal.pone.0027870

**Published:** 2011-11-17

**Authors:** Imke Steffen, D. Lorne Tyrrell, Eleanor Stein, Leilani Montalvo, Tzong-Hae Lee, Yanchen Zhou, Kai Lu, William M. Switzer, Shaohua Tang, Hongwei Jia, Darren Hockman, Deanna M. Santer, Michael Logan, Amir Landi, John Law, Michael Houghton, Graham Simmons

**Affiliations:** 1 Blood Systems Research Institute, San Francisco, California, United States of America; 2 Department of Laboratory Medicine, University of California San Francisco, San Francisco, California, United States of America; 3 Li Ka Shing Institute of Virology, University of Alberta, Edmonton, Alberta, Canada; 4 Department of Psychiatry, University of Calgary, Calgary, Alberta, Canada; 5 Laboratory Branch, Division of HIV/AIDS Prevention, National Center for HIV/AIDS, Viral Hepatitis, STD, and TB Prevention, Centers for Disease Control and Prevention, Atlanta, Georgia, United States of America; National Institute of Health, United States of America

## Abstract

The gammaretroviruses xenotropic murine leukemia virus (MLV)-related virus (XMRV) and MLV have been reported to be more prevalent in plasma and peripheral blood mononuclear cells of chronic fatigue syndrome (CFS) patients than in healthy controls. Here, we report the complex analysis of whole blood and plasma samples from 58 CFS patients and 57 controls from Canada for the presence of XMRV/MLV nucleic acids, infectious virus, and XMRV/MLV-specific antibodies. Multiple techniques were employed, including nested and qRT-PCR, cell culture, and immunoblotting. We found no evidence of XMRV or MLV in humans and conclude that CFS is not associated with these gammaretroviruses.

## Introduction

Chronic fatigue syndrome (CFS), also commonly referred to as myalgic encephalomyelitis (ME), is a complex disorder with an unknown etiology which is characterized by disabling physical and mental fatigue and pain that lasts for at least 6 months and lacks any obvious cause [1], [2]. The sudden onset of symptoms and underlying activation of inflammatory pathways suggest an infectious agent as the triggering factor. Numerous viral and non-viral pathogens have been investigated in the context of CFS with as yet inconclusive results [1], [2]. The xenotropic murine leukemia virus (MLV)-related virus (XMRV) was initially identified in human prostate cancer cells in 2006 [3]. It has since been thought to be the only member of the gammaretrovirus family known to infect humans and its possible role in the development of prostate cancer has been widely suppsed [4]. In 2009, Lombardi *et al.* reported the detection of XMRV in both peripheral blood mononuclear cells (PBMC) and plasma of 67% of a CFS patient cohort compared to 3.7% in healthy controls [5]. This study has gained a high level of attention and was thought to mark a possible break-through in CFS research. Several studies have since addressed the possible connection between XMRV infection and CFS or prostate cancer, and the resulting evidence is controversially discussed in the field [4]. While one study reported the presence of other MLV-like sequences in CFS patients [6], others identified mouse DNA, human cell lines or commercial laboratory reagents to be a possible source of MLV contamination [7]. Attempts to reproduce the initial findings in different CFS patient groups world-wide and in parts of the initial cohort have since failed [4], [8], [9]. Thus, more research is needed to resolve an association of MLV-like viruses in humans. In this study we performed an extensive analysis of whole blood and plasma samples from two well-characterized Canadian CFS patient cohorts and healthy controls utilizing multiple laboratory techniques, including nested and qRT-PCR, cell culture, and immunoblotting for the detection of XMRV/MLV nucleic acids, infectious virus, and XMRV/MLV-specific antibodies.

## Materials and Methods

### Ethics statement

All study protocols were reviewed and approved by the Human Research Ethics Boards of the University of Calgary and the University of Alberta and all study participants provided written informed consent. Laboratory testing of the samples was performed anonymously and blinded.

### Cohorts

All patients and controls examined in this study were part of cohorts from either Calgary or Edmonton, recruited in 2010 and 2011, respectively. All participants completed the De Paul Questionnaire [10] to gather demographic data and to elicit the Canadian Consensus Criteria (CCC) for ME/CFS as established by Carruthers *et al.* [1]. Moreover, all participants were screened according to the Fukuda criteria [2]. Two participants did not meet the CCC and one participant did not meet Fukuda criteria, but all three were included on clinical grounds. The remainder of the CFS group met both the CCC and the Fukuda criteria. Healthy controls who showed more than one symptom of ME/CFS at moderate or greater severity were excluded. The CFS group (58 individuals) had a mean age of 48.9±10.1 years and 90% were female, compared to the healthy control group (57 individuals) with a mean age of 47.6±10.6 years and 89% female, reflecting the higher prevalence of the disease amongst women. A documented infectious onset could be reported by 59% of the CFS patients. Of the CFS patients, 93% have been sick for more than 2 years and 3% have been sick for 1–2 years, while 5% showed symptoms since childhood or adolescence.

### Nested RT-PCR

For detection of XMRV/MLV sequences by nested PCR, RNA was extracted from 0.5 ml plasma using the QIAamp Ultrasens Virus Kit (Qiagen). The isolated RNA was immediately subjected to reverse transcription employing the Superscript III First-Strand Synthesis System for RT-PCR (Invitrogen). Culture supernatant from the XMRV-producing prostate cancer cell line 22Rv1 was used at a 10^−5^ dilution as a positive control for RNA isolation. For amplification of XMRV/MLV *gag* sequences, 5 µl of the transcribed cDNA were used for the first round of amplification with primers 419F (5′-ATCAGTTAACCTACCCGAGTCGGAC-3′) and 1154R (5′-GCCGCCTCTTCTTCATTGTTCTC-3′) [5] and HotStart-IT FideliTaq Master Mix (USB) with the recommended component volumes. The amplification was initiated by incubation for 4 min at 94°C, followed by 40 cycles of 1 min at 94°C, 1 min at 57°C and 1 min at 72°C, and a final incubation for 10 min at 72°C. Nested PCR was performed under the same conditions for 45 amplification cycles with 5 µl of the first round PCR product and two different primer pairs, Gag-I-F (5′-TCTCGAGATCATGGGACAGA-3′) and Gag-I-R (5′-AGAGGGTAAGGGCAGGGTAA-3′) or NP116 (5′-CATGGGACAGACCGTAACTACC-3′) and NP117 (5′-GCAGATCGGGACGGAGGTTG-3′), both of which have been shown to detect both XMRV and MLV sequences [6]. To determine the assay sensitivity, serial dilutions of a cloned fragment of XMRV *gag* [9] ranging from 1 to 100 copies/ µl were included in each PCR. The resulting PCR amplification products (730 bp for first round PCR and 413 bp or 380 bp for second round PCR, respectively) were analyzed by electrophoresis in 1.5% agarose gels. Any bands of approximately the correct size were excised and subjected to sequencing in order to determine homology to MLVs.

### qRT-PCR

**Figure 1:**
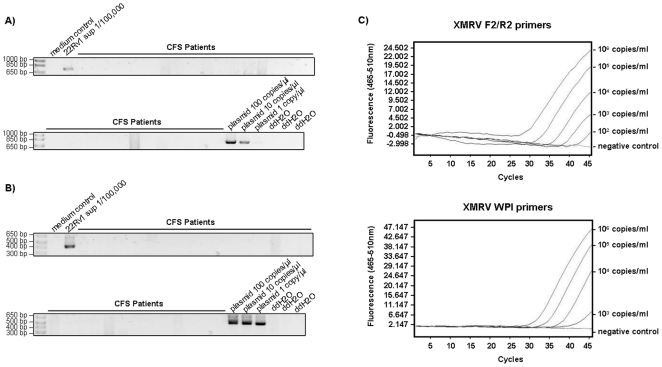
Failure of detection of XMRV nucleic acids in plasma and whole blood of CFS patients and healthy controls. *A)* First round PCR products of a representative number of RNA samples isolated from patient plasma using primers 419F and 1154R. A 10^−5^ dilution of 22Rv1 cell culture supernatant and three known concentrations of XMRV plasmid DNA were included as controls. *B)* Second round amplification products of nested PCR using primers Gag-I-F and Gag-I-R of samples shown in A). Identical results were obtained with primers NP116 and NP117 (see text, data not shown). The detection limit was below 1 copy/ µl isolated RNA or 5 copies/reaction. *C)* Results of qRT-PCR for XMRV plasmid control in serial dilutions ranging from 10^6^ to 10^2^ copies/ml as well as negative controls for both primer pairs used, F2/R2 (upper panel) and WPI (lower panel). All patient plasma and whole blood samples were found to be negative after a total of 45 amplification cycles (data not shown).

For qRT-PCR analysis, RNA was extracted from 100 µl of either whole blood or plasma using the Qiagen Viral RNA Mini Kit. The isolated RNA was subjected to reverse transcription by murine leukemia virus (MuLV) reverse transcriptase (Roche). The resulting cDNA was amplified in a real-time PCR reaction and quantified in a Roche LightCycler 480. Two different primer and probe sets were used for amplification of two distinct regions of the XMRV genome: primers XMRV-F2 5′-AACCTGATGGCAGATCAAGC-3′ and XMRV-R2 5′-CCCAGTTCCCGTAGTCTTTTGAG-3′ and probe FAM-AGTTCTAGAAACCTCTACACTC-BHQ1 for amplification of the XMRV integrase gene [11], and WPI primers Q445F 5′-GGACTTTTTGGAGTGGCTTTGTT-3′ and Q528R 5′- GCGTAAAACCGAAAGCAAAAAT-3′ and probe FAM-ACAGAGACACTTCCCGCCCCCG-BHQ1 for amplification of the XMRV-specific *gag* leader sequence [12] with FastStart Taq polymerase (Roche) in 45 amplification cycles of 95°C and 60°C for 30 sec each. Serial dilutions of a cloned fragment of XMRV *gag* [9] were used to produce standard curves (Fig. 1C). The sensitivity of the qRT-PCR assay was below 10^3^ copies/ml plasma or whole blood.

### Virus culture

DERSE (Detectors of Exogenous Retroviral Sequence Elements) indicator cells were developed at the National Cancer Institute by stable transfection of pBabe.iGFP-puro into LNCaP cells. pBabe.iGFP-puro is an MLV vector encoding puromycin resistance and a CMV promoter driven GFP reporter gene which is interrupted by an intron placed in sense direction relative of the vector and transcribed antisense to the vector mRNA. The intron interrupted GFP gene is only expressed after mobilization by an infecting gammaretrovirus for a second round of infection. After screening clonal cell populations, the most sensitive clones were chosen and designated as DERSE.Li-G cells. To test for the presence of infectious MLVs in patient plasma, DERSE.Li-G cells were inoculated with CFS patient plasma or control plasma. Cells were seeded 72 hours before infection with 3×10^4^ cells/ml in 6-well plates. For spinoculation, the medium was removed and 300 µl fresh medium and 50 µl plasma were added per well. The plates were centrifuged at 1,200 rpm for 1 hour and 0.5 ml fresh medium was added. The inoculum was removed the next day and the cells were cultured in 2 ml fresh medium and monitored for GFP expression every 3 to 4 days for a total period of 3 weeks. As a positive control, culture supernatant from the 22Rv1 cell line (containing roughly 10^9^ copies/ml as determined from the average of seven individual qPCR assays, data not shown) was used as an inoculum at 10^−4^, and 10^−6^ dilution, respectively.

### Serology

**Figure 2:**
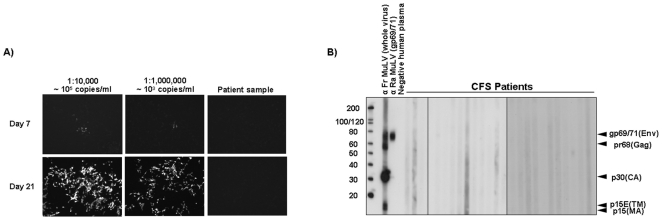
No evidence for infectious virus or XMRV-specific antibodies in plasma of CFS patients and healthy controls. *A)* GFP expression of DERSE.Li-G cells 7 days (upper panels) or 21 days (lower panels) after spinoculation with two different dilutions of 22Rv1 cell culture supernatants (10^−4^ and 10^−6^ dilution) or patient plasma. No GFP expression could be observed in any of the cells inoculated with human plasma. *B)* Immunoblotting of C7-purified XMRV antigen with patient plasma for detection of anti-XMRV/MLV antibodies. Representative WB results for CFS patients and healthy controls. Lane 1, anti-Friend MuLV whole virus, goat polyclonal antisera; lane 2, anti-Rauscher MuLV envelope, goat polyclonal antisera; lane 3, XMRV negative blood donor plasma. Locations of reactivity to specific viral proteins are indicated; Env (gp69/71), envelope; TM (p15E), transmembrane; MA (p15), matrix; Gag (pr68); CA (p30), capsid.

Western blot (WB) analysis was performed to detect anti-XMRV/MLV antibodies in CFS patient sera and healthy controls. Purified XMRV antigen from XMRV-infected DU145 prostate cells (C7) was denatured with SDS-PAGE sample buffer at 95°C for 10 min and analyzed by immunoblotting as previously described [9]. Seroreactivity was defined by reactivity to viral Env and/or Gag proteins of the expected size as seen in the positive control antisera (Fig. 2B).

## Results

Whereas XMRV *gag* sequences were readily detectable in diluted 22Rv1 cell supernatants, XMRV and MLV were not detected in any of the patient plasma samples (Fig. 1A and B). The detection limit of the nested PCR assay was below 1 copy/ µl isolated RNA or 5 copies/reaction as determined by the detection of known amounts of XMRV plasmid DNA (Fig. 1B). The sensitivity of the qRT-PCR assay was below 10^3^ copies/ml plasma or whole blood. Regardless of whether whole blood or plasma was tested, all human samples were negative for detectable amounts of XMRV nucleic acid (data not shown).

DERSE.Li-G cells inoculated with 22Rv1 supernatants showed a concentration-dependent GFP expression on day 7 and spread of the virus on day 21. GFP expression was not observed in any of the DERSE.Li-G cells inoculated with patient plasma (typical example shown in Fig. 2A).

Seroreactivity was defined by Western blot reactivity to viral Env and/or Gag proteins of the expected size as seen in the positive control antisera (Fig. 2B). None of the 115 human plasma reacted with the purified XMRV antigen indicating an absence of antibodies to XMRV/MLV in the samples (typical example shown in Fig. 2B). Increased background noise as observed for one of the CFS patient samples (lane 5, Fig. 2B) is most likely due to the presence of cross-reactive epitopes.

## Discussion

In summary, we were unable to detect any evidence of XMRV or MLV infection in any of the 115 examined study participants, regardless of whether they were suffering from CFS or represented healthy controls. The 58 CFS patients enrolled in this study were carefully selected according to the Canadian Consensus Criteria for ME/CFS. Positively screened participants were only included if they showed symptoms in at least two categories of autonomous, neuroendocrine, and immune manifestations. The sensitivity of our assays reached copy numbers lower than 120 copies/ml of plasma for the detection of viral nucleic acids, and 10^3^ copies/ml of plasma for the presence of infectious particles. While it is possible that XMRV and MLV are not predominantly blood-borne viruses and as such exist below the detection limit of most assays in plasma and whole blood, we believe that the assays used in this study are equally sensitive to those reported in previous positive studies. Moreover, our broad study design and the use of degenerate primers with specificity for highly conserved sequences in different MLV-like viruses and XMRV would have allowed us to identify nucleic acids, infectious particles, and antibodies for a number of related murine retroviruses. However, we could not detect any other murine retroviruses in any of our specimens, unlike the finding of MLV-like sequences reported by Lo *et al.* [6].

CFS patient cohorts have been tested for the presence of XMRV in the United States, Netherlands, Germany, China, and United Kingdom among others [4]. Being more aware of the possible risk of contaminants in commonly used laboratory reagents [13], none of these studies were able to reproduce the initial findings. Moreover, repeated testing of CFS patients previously reported to be infected with XMRV in the initial study performed by Lombardi *et al.* failed to detect any signs of XMRV infection in these patients [8]. On the contrary, it is now becoming increasingly clear that XMRV found in the prostate cancer cell line 22Rv1 originated from recombination of two MLVs present in the mouse strains used for passaging of the initial prostate cancer xenograft [14]. The fact that the viral sequences initially identified in prostate and CFS samples are virtually identical to those found in 22Rv1 cells [15] suggests that the assumed association of XMRV with human diseases is due to sporadic laboratory contamination. Moreover, differential handling of patient samples compared to controls can introduce bias and was therefore carefully avoided in this study. Two independent studies could show that handling of human samples in laboratory environments with abundant endogenous MLV proviruses can lead to the false detection of XMRV/MLV-like sequences due to contamination as proven by PCR detection of the highly abundant intracisternal A-type particle (IAP) long terminal repeat in the same samples [16], [17]. In the light of the accumulating evidence for the artefactual origin of XMRV and the high burden of MLV-like DNA contamination the initially reported connection of XMRV and prostate cancer is now being ruled out as well [18]. Thus, although XMRV was found to infect and replicate in a variety of human cells, natural XMRV/MLV infection of humans has not yet been reproduced and is believed to be a false-positive result from mouse DNA and/or MLV-contaminated PCR reagents [13]. This study examines a possible association of XMRV and chronic fatigue in a Canadian patient cohort and is consistent with a number of recently published reports declaring no evidence for the presence of MLV-like viruses in any human subjects. In conclusion, while this study and others fail to support an association between XMRV and CFS, they highlight the urgent need for further research into the root causes of CFS.
